# The Consequences of GBA Deficiency in the Autophagy–Lysosome System in Parkinson’s Disease Associated with GBA

**DOI:** 10.3390/cells12010191

**Published:** 2023-01-03

**Authors:** Eddie Pradas, Marta Martinez-Vicente

**Affiliations:** 1 Neurodegenerative Diseases Research Group, Vall d’Hebron Research Institute-CIBERNED, 08035 Barcelona, Spain; 2 Protein Engineering and Nanomedicine Group, Institut de Biotecnologia i Biomedicina, Universitat Autònoma de Barcelona, 08193 Cerdanyola del Vallès, Spain

**Keywords:** GBA, Parkinson’s disease, autophagy, lysosome, alpha-synuclein

## Abstract

*GBA* gene variants were the first genetic risk factor for Parkinson’s disease. *GBA* encodes the lysosomal enzyme glucocerebrosidase (GBA), which is involved in sphingolipid metabolism. GBA exhibits a complex physiological function that includes not only the degradation of its substrate glucosylceramide but also the metabolism of other sphingolipids and additional lipids such as cholesterol, particularly when glucocerebrosidase activity is deficient. In the context of Parkinson’s disease associated with GBA, the loss of GBA activity has been associated with the accumulation of α-synuclein species. In recent years, several hypotheses have proposed alternative and complementary pathological mechanisms to explain why lysosomal enzyme mutations lead to α-synuclein accumulation and become important risk factors in Parkinson’s disease etiology. Classically, loss of GBA activity has been linked to a dysfunctional autophagy–lysosome system and to a subsequent decrease in autophagy-dependent α-synuclein turnover; however, several other pathological mechanisms underlying GBA-associated parkinsonism have been proposed. This review summarizes and discusses the different hypotheses with a special focus on autophagy-dependent mechanisms, as well as autophagy-independent mechanisms, where the role of other players such as sphingolipids, cholesterol and other GBA-related proteins make important contributions to Parkinson’s disease pathogenesis.

## 1. Introduction

Autophagy involves the lysosome-associated degradation and recycling of intracellular elements, including proteins, organelles and other components. Autophagy is an evolutionarily conserved mechanism that is essential for the maintenance of cellular homeostasis through the continuous turnover of the cellular proteome and the selective removal of malfunctioning proteins and organelles [1].

In mammalian cells, three major forms of autophagy have been described: macroautophagy (MA), chaperone-mediated autophagy (CMA) and microautophagy. These autophagy types comprise different and coexisting pathways through which intracellular material is delivered to lysosomes for degradation, allowing the disassembly of macromolecules and the recycling of their constituents [2].

Malfunctioning of the autophagy pathways and lysosomal dysfunction has been reported in several diseases, including neurodegenerative disorders such as Parkinson’s disease (PD) [3]. Under normal physiological conditions, active neuronal autophagy controls the turnover of organelles and proteins and prevents the conversion of neurotoxic proteins associated with neurodegenerative diseases, such as α-synuclein, from their native conformations to fibrillary aggregates.

Several studies have shown that the genetic or pharmacological blockage of autophagy leads to the accumulation of aggregates and is sufficient to cause neurodegeneration [4,5,6,7,8]. In this context, the functional decline of autophagy during aging [2] has been proposed to be a primary risk factor for neurodegenerative diseases.

Hence, impaired autophagy caused by genetic mutations, environmental factors or aging leads to a reduction in the ability to remove damaged organelles and pathogenic proteins, contributing to the formation of the protein aggregates observed in affected regions of the central nervous system (CNS) in different neurodegenerative diseases, such as Alzheimer’s disease, PD, Huntington’s disease and amyotrophic lateral sclerosis [9,10].

In this review, we focus on autophagy–lysosome system dysfunction caused by mutations in the *GBA* gene, the main genetic risk factor associated with PD. In summary, we summarize the GBA-dependent mechanisms that can promote alterations in the autophagy–lysosome system and contribute to PD pathogenesis.

## 2. Parkinson’s Disease and GBA

### 2.1. Parkinson’s Disease and the Lysosomal System

PD is the second most common neurodegenerative disease after Alzheimer’s disease, affecting 2–3% of individuals over 65 years of age. PD is clinically characterized by motor symptoms such as restful tremor, rigidity, bradykinesia and postural instability as well as by nonmotor symptoms including neuropsychiatric alterations, autonomic dysfunction, sleep disorders and hyposmia [11,12]. The disease is characterized by the selective and progressive loss of dopaminergic neurons, mainly in the substantia nigra pars compacta (SNpc), and in other regions, such as the locus coeruleus (LC) [13], and by intraneuronal inclusions called Lewy bodies (LB) [14]. A LB is a complicated complex formed mostly by aggregated proteins, with α-synuclein as the best-characterized protein and main LB marker [15]. Notably, LBs also include nonproteinaceous materials such as lipids and membranous organelles [16]. 

Because α-synuclein can self-assemble, oligomerize and form fibrils, α-synuclein levels and conformations are considered to play central roles in PD pathogenesis [17]. The progressive accumulation of α-synuclein neurotoxic species (i.e., oligomers, protofibrils and fibrils) in the SNpc characterize PD as a synucleinopathy, a disease category that includes other neurodegenerative disorders such as dementia with Lewy bodies (DLB), in which α-synuclein aggregates accumulate mostly in the frontal cortex, and multiple system atrophy (MSA), in which they accumulate in oligodendrocytes, forming glial cytoplasmic inclusions [18]. 

The multifactorial etiology of PD involves different factors; however, among them, aging remains the major risk factor for developing PD, in addition to environmental factors and genetic predisposition. Recent advances in genetic research have significantly improved our understanding of PD. In fact, several genes have been associated with both autosomal dominant and recessive forms of PD [19,20], and several risk genes for sporadic PD have been identified by genome-wide association studies [21,22,23,24]. 

With relevance to this review, a meaningful number of PD-associated genes, including *GBA*, *LRRK2*, *ATP13A2*, *TMEM175*, *VPS35*, *ATP6P2*, *RAB7L1*, *VPS13C*, *DNALC13*, *DNAJC6*, *PINK1*, *PRKN*, *UCHL-1* and *CTSD* are involved in the autophagy–lysosome–endosomal system [25]. This association highlights the relevant involvement of these pathways in PD pathogenesis. Among all these genes, *GBA* is currently considered the most important genetic risk factor for PD [26] because 5–30% of PD patients (depending on the ethnicity of the population) present with *GBA* mutations, and *GBA* mutations are much more frequent than mutations of other genes typically associated with familial PD, such as SNCA or LRKK2 [27,28,29].

### 2.2. Parkinson’s Disease Associated with GBA

Biallelic mutations in the *GBA* gene cause autosomal recessive Gaucher’s disease (GD), the most common lysosomal storage disorder (LSD). GD is characterized by a decrease in glucocerebrosidase (GCase) activity and the subsequent accumulation of its sphingolipid substrate glucosylceramide (GlcCer) and, at even higher abundance, the deacetylated derivative of GlcCer, glucosylsphingosine (GlcSph). These sphingolipids accumulate in several organs, mostly the bone marrow, liver and spleen, but also in the CNS in patients presenting with the neuropathic forms of GD [30]. 

Homozygous and heterozygous *GBA* mutations confer an important and increasing risk of developing PD; that is, heterozygous carriers present a cumulative risk of developing PD of approximately 5% at age 60 years, which increased to 15–30% at age 80 years [31,32,33].

*GBA* mutations are present in 5–30% of PD patients according to the ancestry of the population [34]. The neuropathological markers of PD associated with GBA mutations (PD-GBA) are the same as those associated with idiopathic Parkinson’s disease (iPD); also presenting dopaminergic cell loss and LB pathology. Moreover, PD-GBA patients show an earlier age of onset than iPD patients with more rapid progression, increased involvement of cognitive functions and more-severe motor and nonmotor symptoms and autonomic dysfunction [35,36,37,38,39].

According to recent studies with large cohorts, the risk of developing PD and the severity of the clinical features of these patients (age at onset, motor phenotype, neuropsychiatric symptoms, cognitive dysfunction, mortality, etc.) may be associated with the severity of *GBA* mutation. Therefore, although more than 300 *GBA* gene variants have been described, these variants can be stratified as risk variants (e.g., p.E326K and p.T369M), mild risk variants (e.g., p. N370S and p. R496H) and severe risk variants (e.g., p.L444P, p.D409H, p.V394L and RecTL) [33,40,41].

In addition to PD, increased risk associated with *GBA* variants has been analyzed in other synucleinopathies. *GBA* mutations have been positively associated with DLB and Parkinson’s disease dementia (PDD), but to confirm the association between *GBA* mutations and MSA, further investigation is needed [42,43,44,45,46].

## 3. The *GBA* Gene Encodes the β-Glucocerebrosidase Enzyme

The *GBA* gene, located on chromosome 1q21, comprises 10 introns and 11 exons. The *GBA* gene carries two distinct promoters that are differentially activated depending on the cell type. Of special interest in this review, the P2 promoter has been shown to harbor CLEAR domains, which are recognized by the master regulator of lysosomal biogenesis, the transcriptional factor EB (TFEB) [47]. Interestingly, the *GBA* gene carries a pseudogene (*GBAP*) adjacent to it. *GBAP* shows 96% homology with *GBA*, but lacks translational capacity, although its mRNA has been proposed to be a competing endogenous RNA (ceRNA) that functions as an microRNA sponge, leading to a higher *GBA* gene translation rate [48,49].

The *GBA* gene encodes β-glucocerebrosidase (GCase), i.e., the GBA protein, a lysosomal enzyme whose main catalytic function is the hydrolysis of GlcCer into ceramide and glucose. The enzymatic function of this enzyme is pH dependent, with an optimal pH of 5.5, corresponding to the lysosomal pH [50]. The GBA protein comprises three structural domains: domain I (residues 1–27 and 383–414), consisting of an antiparallel β-sheet with two disulfide bridges, whose function is thought to be structural; domain II (residues 30–75 and 431–497), which is an immunoglobulin-like structure, usually considered to be an interaction domain; and domain III (residues 76–381 and 416–430), which is the catalytic domain with a TIM barrel structure [51]. The structural features of GBA have been reviewed extensively in [52]. 

**Figure 1:**
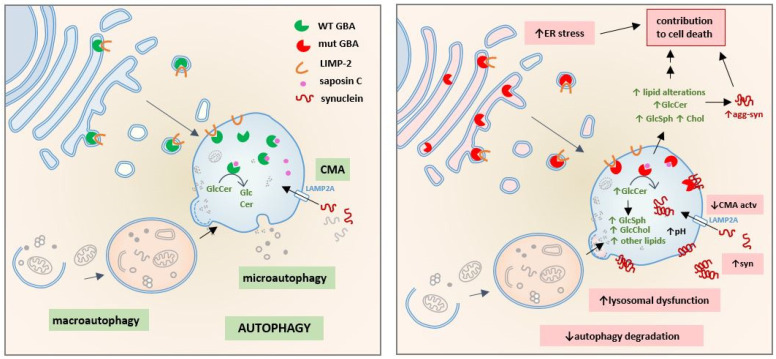
Consequences of GBA deficiency in the autophagy-lysosome system: GBA is synthesized in the ER, recognized by its specific transporter LIMP-2, and both are transported to the lysosome where the GBA-LIMP-2 complex dissociates and GBA interacts with its coactivator saposin C. GBA hydrolyzes GlcCer to ceramide and glucose. Under physiological conditions, CMA is the major proteolytic pathway to degrade soluble synuclein (**left**). GBA mutations (**right**) activate different pathogenic mechanisms that contribute to cell death; mutant GBA is partially retained in the ER, generating ER stress. Loss of lysosomal GBA activity leads to accumulation of GlcCer and triggers the abnormal increase of other lipids such as GlcSph, cholesterol and gangliosides inside and outside the lysosome. In the lysosome, these changes in lipid metabolism can induce CMA blockade, increase synuclein accumulation, promote synuclein-membrane interaction, increase lysosomal pH and lead to lysosomal dysfunction. As a consequence, autophagy degradation is also impaired since lysosomes are the terminal compartment where all autophagy pathways deliver intracellular components to be degraded.

As a lysosomal protein, GBA is synthetized within the ER, and after its translation, GBA is recognized by its specific transporter LIMP-2 [53,54]. Both GBA and LIMP-2 are transported to their final destination: a lysosome [55] (Figure 1). The stability and trafficking of the GBA–LIMP-2 complex is a pH-dependent process; that is, the interaction between GBA and LIMP-2 is favored at a neutral pH but disfavored at an acidic pH, and therefore, GBA–LIMP-2 binding is stable during trafficking through the ER and Golgi, but the complex dissociates upon entry into the acidic environment of the lysosomal lumen [56]. Studies performed to characterize the GBA–LIMP-2 complex have demonstrated that a lack of interaction with LIMP-2 leads to the almost total abrogation of GBA activity and mislocalization to the ER or extracellular milieu [54,57,58].

Once at a lysosome, GBA interacts with its coactivator saposin C (Sap C). The GBA–Sap C complex is critical for GlcCer hydrolysis. The binding of Sap C to GBA seems to decrease the pH from that of optimal GBA activity (pH 5.5) to a more acidic one at pH 4. Furthermore, it has been demonstrated that the complex is markedly more active in the presence of negatively charged membranes [59,60,61,62]. Related to PD, studies have shown that the GBA N370S mutant exhibits diminished capacity to bind Sap C and negatively charged membranes, suggesting a reason for the decreased catalytic activity of the mutant [63].

## 4. Lipid Metabolism Alterations Associated with GBA Deficiency

### 4.1. Sphingolipid Alterations

**Figure 2:**
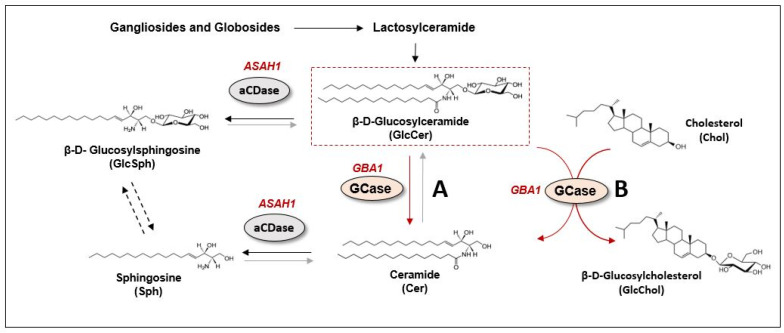
GBA-related lipid metabolism. β-D-Glucosylceramide (GlcCer) is cleaved by glucocerebrosidase enzyme (GCase or GBA, encoded by GBA1 gene) to generate free ceramide (Cer) (A reaction in red). In a healthy condition, there is a fast hydrolysis of GlcCer into Cer, which is then converted to sphingosine (Sph) by acid ceramidase enzyme (aCDase, encoded by ASAH1 gene) (reaction in black). In absence of GBA/GCase activity, GlcCer cannot be converted into Cer and its conversion to β-D- Glucosylsphingosine (GlcSph) by aCDase is favored The direct conversion of GlcSph into Sph is still under debate (reaction in dotted black). GBA/GCase secondary enzymatic activity in the transglycosidation reaction between GlcCer and cholesterol (Chol), producing Cer and glucosylated cholesterol (GlcChol) (B reaction in red). This reaction has been shown to act in both directions, the inverse reaction being more physiologically relevant.

Metabolically, GBA is the enzyme critical for the last step in the catabolism of most glycosphingolipids, including gangliosides and globosides. Complex glycosphingolipids are modified by sugars via the actions of multiple enzymes to ultimately generate GlcCer. Then, GlcCer is cleaved by GBA to generate free ceramide (Figure 2). As previously mentioned, in GD, the loss of enzymatic activity causes the profound accumulation of GlcCer, predominantly in macrophages called Gaucher cells [64]. However, lipid alterations in GD are not limited to excessive GlcCer accumulation; they also affect different points in sphingolipid and glycosphingolipid metabolic pathways [65,66,67]. Indeed, under lipid-altering conditions, accumulated GlcCer is naturally deacetylated to generate GlcSph (Figure 2), also known as lyso-Gb1, which is used in clinical practice because it is the most sensitive and specific biomarker for the diagnosis and monitoring of patients with GD [68,69,70].

In a PD-GBA background, where GBA mutations appear during heterozygosis and where its enzymatic activity is partially maintained, lipid accumulation is not as clear as it is in homozygous mutants. Nonetheless, although several studies failed to find significant changes in sphingolipid levels, other studies reported alterations in the levels of specific lipids, such as GlcCer, GlcSph, sphingosine (Sph) and sphingosine-1-phosphate (S1P), or complex glycosphingolipids, such as the gangliosides GM1 and GM3, in patient-derived samples, including serum, cerebrospinal fluid (CSF) and postmortem brain tissue. For a detailed summary, please see the extensive review in [71]. In addition to alterations due to GBA mutations, the aging process in healthy subjects produces a marked decrease in GBA activity and lipid accumulation. These alterations are more pronounced in the SNpc and the putamen, areas in the brain related to PD [72]. 

Although the literature on glycosphingolipid accumulation or ceramide metabolism alterations in the context of PD-GBA has been inconclusive, several considerations are needed when making conclusions on the basis of these experimental data. First, the extent of lipid accumulation differs by cell type; for example, in a postmortem analysis of homogenate samples from the whole brain, the lipidomic results of the total tissue can mask an alteration in a specific cell type, such as a neuron. Second, the abnormal accumulation of these lipids in cells can be exacerbated in particular subcellular compartments, such as lysosomes, as shown in an in vitro PD-GBA neuronal model, where the pronounced accumulation of glycosphingolipids was detected in the lysosomal compartment, while analysis of whole-cell lipids showed small or negligible accumulation of these lipids [73]

Regarding the relationship of glycosphingolipids with autophagy, some studies have pointed to GlcSph but not GlcCer as a toxic byproduct of GBA dysfunction that alters, among other pathways, autophagic signaling pathways [74]. Under physiological conditions, GlcSph is not detected in cells since GlcCer is cleaved into glucose and ceramide by GBA under basal conditions. The ceramide generated is thus deacylated by acid ceramidase, producing Sph and fatty acids. However, under GBA-deficient conditions, where ceramide cannot be generated from GlcCer, the substrate for acid ceramidase becomes GlcCer, and thus, GlcSph, a toxic byproduct, is generated [75,76,77] (Figure 2). The accumulation of GlcCer and GlcSph has been directly related to many deleterious mechanisms in cells, and several of these mechanisms affect the autophagy–lysosome system. For example, increased levels of such lipids are related to altered lysosomal pH [78,79], altered membrane trafficking [80], CMA dysfunction [73] and also the hyperactivation of mTORC1, the master regulator of autophagy, leading to an MA inhibition [74] (Figure 1). 

Supporting this hypothesis, the pharmacological reduction of these sphingolipid levels by the inhibition of upstream enzymes in the metabolic pathway of GlcCer formation or the enzyme producing toxic GlcSph can partially correct these multiple defects [64,74,81,82]. 

### 4.2. GBA and Lysosomal Cholesterol Metabolism

Lysosomes are not merely degradative and recycling organelles; they also have an important role in cell metabolism as nutrient-sensing and metabolic signal transduction hubs [83]. These functions are coordinated by mTORC1 on the lysosomal surface [84,85]. In response to different metabolic signals, such as nutrient, ATP or cholesterol levels, mTORC1 can trigger the activation/inhibition of several cellular pathways, including autophagy pathways [86,87].

In this context, lysosomes play an important role in cholesterol metabolism [88] and GBA deficiency can affect lysosomal cholesterol metabolism, as evidenced in different cell and animal models where high levels of lysosomal cholesterol have been described under GBA-deficient conditions [63,73,89].

In addition to the well-characterized activity of the GBA protein in the catabolism of glucosylceramide, GBA shows other types of enzymatic activity, such as in the transglycosidation reaction between GlcCer and cholesterol, producing ceramide and glucosylated cholesterol (GlcChol) [90,91,92]. Notably, this reaction can work both ways, showing that GBA is also able to degrade GlcChol to generate free cholesterol and glucose (Figure 2). In fact, cellular studies on GlcChol synthesis have shown that the inhibition of GBA was accompanied by an increase in GlcChol level, while the inhibition of GBA2 (nonlysosomal glucocerebrosidase located in the ER) led to a reduction in GlcChol level [91]. These results suggest that under physiological conditions, GlcChol is generated by GBA2, while GBA may also hydrolyze GlcChol to generate free cholesterol.

Moreover, not only GBA deficiency can affect lysosomal cholesterol, but other GBA-related proteins are also involved in cholesterol metabolism. In addition to its function as a GBA transporter, LIMP-2 has been shown to be a cholesterol transporter. LIMP-2 shares high homology with SR-BI, a protein related to cholesterol export from low-density lipoproteins (LDLs), and this activity has also been reported for LIMP-2 [93,94,95]. This cholesterol export function seems to be coupled to NPC1, the key lysosomal cholesterol exporter. Indeed, NPC1 seems to be related to GBA and LIMP-2, as NPC1 dysfunction negatively affected GBA enzymatic activity and LIMP-2 function, demonstrating reciprocity between these systems [96] Additionally, alterations in cholesterol transport found under NPC1-deficient conditions were accompanied by glycosphingolipid and Sph accumulation inside lysosomes, disrupting endosomal trafficking and increasing lysosomal pH, similar to the effects of GBA deficiency [89].

Dysregulated levels of lysosomal cholesterol can affect autophagy machinery at several steps. In the lysosomal context, the mechanistic regulation of mTORC1 activity via changes in lysosomal cholesterol levels has been established. SLC38A9 is a lysosomal transmembrane protein and a component of the lysosomal amino acid sensing machinery that controls the activation of the mTORC1 controlling the uncoupling of the Ragulator complex from v-ATPase at the lysosomal surface [97]. In the presence of cholesterol, SLC38A9 allows the disassembling of the Ragulator complex from v-ATPase and the consequent activation of mTORC1 resulting in the inhibition of MA initiation. In contrast, in the absence of cholesterol, Ragulator is sequestered by v-ATPase through SLC38A9, impeding mTORC1 activation and promoting autophagic induction [87]. In this context, NPC1 exporting cholesterol from the lysosome plays a role as an inhibitor of mTORC1 that leads to autophagy activation [97]. Analogously, CMA can also be modulated by abnormal levels of lysosomal cholesterol that favor the degradation of LAMP-2A proteins at the lysosomal membrane and diminish CMA activity [73,98,99].

## 5. α-Synuclein Metabolism

### 5.1. α-Synuclein Protein

α-Synuclein is an unstructured 140 amino acid protein comprising three domains. The N-terminal domain (residues 1–60) carries a multiply repeated consensus sequence (KTKEGV) and shows the propensity to form an alpha-helix. The central domain (residues 61–95), called the non-amyloid-β component (NAC), is highly hydrophobic and critical for the β-sheet conformation of aggregated α-synuclein. The C-terminal domain (residues 96–140) is enriched in negatively charged residues and is involved in protein recognition and interaction [100]. α-Synuclein has been classically described as a totally unstructured protein in solution. Nonetheless, this intrinsically unstructured structure is lost upon interaction with lipidic membranes [101].

### 5.2. α-Synuclein Interaction with Lipid Membranes

α-Synuclein binding to lipid bilayers is highly dependent on the lipid composition of the membranes. α-Synuclein can sense the net charge of a head group, binding strongly to negatively charged membranes [102]. In the PD context, multiple studies have shown that α-synuclein shows a marked preference for lipid microdomains [103,104,105]; this preference is guided by two interaction domains in α-synuclein. The first of these domains is located in the N-terminus of the protein. A glycosphingolipid binding domain (GBD) comprises residues 34–45 of the protein. This GBD recognition sequence shows affinity for various gangliosides, but it has a strong affinity for the GM3 lipid. In the presence of GM3, α-synuclein shows the capacity to enter the lipid bilayer through the integration of T39 [106] Insertion of α-synuclein in the lipid bilayer produces α-synuclein channels that act as ion channels. In this context, the α-synuclein E46K mutant, with relevance in PD, exhibits enhanced affinity for GM3. This increased affinity modifies α-synuclein channel selectivity for cations possibly explaining, in part, its toxicity [107]. The second domain is a cholesterol recognition region in the NAC domain. This region is particularly interesting, as cholesterol binding is possible only at a 45° angle with respect to the membrane, which makes this site of interaction similar to a membrane fusion domain [108].

Although the nature of α-synuclein binding to lipid membranes remains to be fully elucidated, the proposed functions of α-synuclein include membrane fusion, membrane shape remodeling and lipid extraction from membranes via a mechanism resembling that of apolipoproteins [103,106,107,109]. In relation to its preferential binding affinity for lipid rafts, α-synuclein has been shown to act as a cholesterol-lowering agent. α-synuclein overexpression either in astrocytic or neuronal cells promotes a decrease in total cholesterol levels [110,111]. 

Other authors pointed to the interaction between α-synuclein and the membrane as a toxicity-inducing mechanism, with the binding sites acting as seeding points for α-synuclein oligomerization and fibrillation, leading to toxicity and ultimately to cellular dysfunction. In this regard, membrane lipid composition seems to modulate α-synuclein aggregation [112,113,114,115]. In a GBA-PD context, GlcCer and GlcSph seem to induce α-synuclein aggregation both in vitro and in vivo [81,116,117] (Figure 1).

### 5.3. Direct GBA–α-Synuclein Interaction

Multiple studies have suggested a direct interaction between GBA and α-synuclein in a lysosomal context. In the first such study, performed in 2011, NMR experiments revealed that the C-terminal region of α-synuclein interacted with GBA at pH 5.5 but not at pH 7 when incubated in solution. Additionally, GBA coprecipitated with α-synuclein in healthy brain samples but this interaction was reduced under mutant GBA conditions despite that higher α-synuclein levels were present under these conditions [118]. Subsequent studies confirmed these data in the presence of negatively charged vesicles demonstrating that α-synuclein favored the interaction of GBA to membranes. Studies have shown that in the absence of GBA, α-synuclein was located at membranes in the form of a double antiparallel alpha-helix. Binding to GBA maintains the first alpha helix at the membrane, but the second helix extrudes from the membrane during α-synuclein interaction with GBA, leaving only residues 1–37 in contact with the membrane [119,120].

Studies analyzing GBA activity upon its interaction with α-synuclein (in the presence of negatively charged vesicles) seemed to indicate that the GBA catalytic pocket is oriented to the membrane, suggesting capacity to interact with its substrates. Nonetheless, in these studies, α-synuclein seemed to inhibit GBA activity. Sap C addition relieved this inhibition by displacing the α-synuclein [121,122]. Although α-synuclein seems to act as a GBA inhibitor in this context, the physiological role of the α-synuclein-GBA interaction remains to be elucidated.

Finally, GBA can affect α-synuclein metabolism through the modulation of the autophagy–lysosome pathway. The following sections summarize our understanding of the major consequences of GBA deficiency on autophagy pathways and α-synuclein turnover.

## 6. GBA Deficiency and Macroautophagy Dysfunction

### 6.1. Macroautophagy in Parkinson’s Disease

MA is the mechanism by which cytoplasmic material is sequestered within double-membraned vesicles named autophagosomes and delivered to lysosomes for degradation. Complex machinery regulates the initiation of MA, autophagosome formation and elongation, cargo recognition and trafficking and fusion with lysosomes [123]. The intracellular material degraded by MA can comprise large portions of the cytoplasm during “bulk” MA but can also be selective, where specific cellular components are targeted for lysosomal degradation. According to the selective cargo recognized and eliminated by MA, different forms of MA have been described: mitophagy, aggrephagy, ER-phagy, ferritinophagy, glycophagy, lipophagy, pexophagy, ribophagy, RN/DNautophagy, xenophagy, etc. [124,125].

As previously mentioned, genetic studies have identified several genes associated with Mendelian and sporadic forms of PD as well as PD risk variants; among these genes, numerous genes, such as Parkin, PINK1 and LRRK2, have been linked to MA pathways [3,25].

### 6.2. Macroautophagy in Parkinson’s Disease Associated with GBA

Although GBA is not directly involved in the MA machinery, in recent years, several works have claimed that the loss of GBA activity is linked to MA dysfunction. One of the main hypotheses suggests that the accumulation of α-synuclein in conjunction with GBA deficiency is related to the deregulation of MA, which impairs the degradation of the high-molecular-weight species of α-synuclein and thus promotes its accumulation. The literature on the effect of GBA deficiencies on the development of MA defects is very diverse; in addition, the studies are performed using several different types of biosamples and cellular or patient-derived biosamples and as a result, most of the outcomes are inconclusive. In this review, we analyze and summarize the controversial results on the basis of the sample and strategy used to analyze the effect of GCase loss (Table 1).

#### 6.2.1. GBA Inhibition by Conduritol-β-Epoxide (CBE)

CBE is a covalent inhibitor of GBA and has been used as a strategy to mimic GBA deficiencies. Studies in vitro using this CBE in differentiated SH-SY5Y cells and rat cortical neurons showed that the LC3-II and α-synuclein levels remain unchanged after CBE inhibition with no alterations in MA or CMA [126]. Nonetheless, later studies using the same cell model showed an increase in LC3-II and p62 levels and accumulation of α-synuclein with a reduction in mTORC1 activity, suggesting a defect in the autolysosome reformation machinery, according to authors [127]. In vivo studies inducing the chronic inhibition of GBA in mice revealed increased LC3-II and p62 levels in the SN with increased proteinase-K-resistant α-synuclein aggregates [128] suggesting that the inhibition of GCase activity impaired MA flux and led to the accumulation of MA markers (Table 1).

#### 6.2.2. Inhibition of GBA Expression

Another strategy that has been used to assess GBA deficiencies is the use of short interfering RNA (shRNA) to induce abrogate GBA expression. Different studies have used short interfering RNA against GBA (siRNA-GBA) in vitro with opposite results but the same interpretation suggests a blockage of the MA flux.

Some works using human neuroblastoma or neuroglioma cell lines showed that knocking down GBA expression led to increases in p62 and LC3-II levels, which were attributed to MA blockage [127,129]. In contrast, other works concluded that MA was inhibited; they showed a reduction in the LC3-II level and in the autophagic flux in SK-N-SH cells and rat cortical neurons treated with shRNA-GBA. According to the authors, the decrease in ceramide due to the loss of GCase activity inactivated PP2A, an indirect inhibitor of the MA-induction inhibitor mTORC1 [130] (Table 1).

#### 6.2.3. GBA-Knockout Models

GBA-KO models have been used to determine the effects of GCase loss in MA. Notably, the results and interpretations of different studies were inconsistent.

In vitro studies with cortical neurons from mouse *GBA*^-/-^ mice showed decreased levels of total LC3 and ATG5/12 (MA-initiating proteins) and accumulation of p62 and aggregated α-synuclein, suggesting impaired MA [131]. In contrast, other in vitro models based on GBA-KO cells showed no significant changes in LC3-II levels in mouse embryonic fibroblasts or BE(2)M17 GBA-KO cells [73]. Other works showed a clear increase in MA markers in the absence of the GBA protein [81], which was attributed to the accumulation of toxic GlcSph. These observations implicate lipid substrates in MA defects in GBA-KO cells, which had also been observed in GBA-KO immortalized neurons. In the latter study, cells showed increased LC3-II and hyperactivated mTORC1 levels. 

Substrate reduction therapy (SRT) targeting and inhibiting glucosylceramide synthase (GCS) reduced the levels of GlcCer and GlcSph. The SRT therapy is currently and successfully used in patients with GD to avoid the accumulation of GBA substrates [147,148] and when used in vitro, authors observed partial restoration of LC3-II levels and reduction in mTORC1 hyperactivation [82].

In vivo, GBA-KO flies showed increases in LC3-II and p62 levels, which were interpreted to be MA failure and lysosomal dysfunction. Treatment with rapamycin ameliorated the GBA-KO phenotype [132] (Table 1).

#### 6.2.4. Mutant GBA Models

In contrast with the experimental pharmacological or genetic inhibition of GBA, several studies aimed to analyze the role played by GBA in PD based on cell and animal models carrying clinically relevant GBA mutations.

These in vitro models were composed of patient-derived cells such as fibroblasts and peripheral blood mononuclear cells (PBMCs), different immortalized cell lines carrying mutant GBA and induced pluripotent stem cells (iPSCs) or neuronal-derived cells.

Fibroblasts derived from PD patients carrying GBA mutations (analyzed in parallel with GD patients and/or idiopathic PD patients) have been broadly used to investigate the effect of GCase activity on lysosomal/autophagic function [89,127,134,135,149]. However, since the genetic program of fibroblasts differs markedly from the genetic program of dopaminergic neurons, analyses with fibroblasts present important limitations to analyze several neuronal features. According to the authors of a study, fibroblasts from neuronopathic GD with a homozygous L444P mutation presented blocked MA flux. This MA blockade seemed to be linked to late stage MA, as the expression of ATG5/12 (MA-initiating proteins) was increased, suggesting that autophagy initiation was not blocked [134]. Another study using N370/WT fibroblasts from a GBA-PD patient revealed increased levels of LC3-II but failed to show blocked autophagic flux. This study also showed altered lysosomal function including higher levels of cholesterol inside the lysosomes and accumulation of multilamellar bodies (MLB) [89].

Studies on peripheral PBMCs from sporadic PD patients showed an increase in the LC3-II mRNA and protein levels, which was related to an increase in MA, probably because of a compensatory mechanism [136]. In line with previously established models, different in vitro immortalized neuronal cells, namely SH-SY5Y and BE(2)M17 cells, carrying GBA mutations showed diverse effects on MA and lysosomal function but both works showed an attempt by the cell to activate MA to compensate for CMA impairment [73,139].

Finally, neurons derived from iPSCs of GD patients or GBA-PD patients have also been shown to be excellent tools for the study of MA defects in cell culture. In 2014, a study with neurons derived from iPSCs of GBA-PD patients (N370S/WT and L444P/WT cells) showed a consistent increase in lysosomal content and basal LC3-II levels [141]. MA blockade was confirmed by a reduction in macroautophagic flux in these cells, an effect that was reversed by treatment with recombinant GBA. In 2015, using neurons derived from iPSCs of neuronopathic GD patients, a similar increase in LC3-II and decrease in autophagic flux were observed [142]. Furthermore, and related to lysosomal dysfunction, a decrease in TFEB expression was identified, with a subsequent decrease in lysosomal biogenesis. These results showing lysosomal function, including an LC3-II increase and autophagic flux decrease, seemed to be consistent, as later studies with this type of cell model showed the same effect. However, these alterations may be related to other cellular events, such as ER stress induced by misfolded proteins stacked in the ER or with the hyperactivation of the mTORC1 pathway [142,150] Furthermore, the presence of enlarged lysosomes and autolysosome structures observed via transition electron microscopy seemed to confirm macroautophagy blockade in the latter step of autolysosome degradation [143,151]. These alterations in the mTORC1 pathway seemed to be related to the accumulation of the toxicity-inducing substrate GlcSph, as the defects were ameliorated by acid ceramidase inhibition (which is the enzyme critical for GlcSph generation upon GlcCer accumulation inside lysosomes). Exogenous GlcSph addition to the cell culture led to phenocopied MA alterations in these cells [74].

In vivo studies using mouse models carrying mutant GBA were focused mostly on the analysis of synuclein levels and lipid alterations. Few studies analyzed MA markers [137,144,145,146]; with the GBA-D409V knock in mouse, most works failed to detect abnormal MA [144,145,146]. Only in the GBA-L444P model, authors detected an alteration in the MA flux, mitophagy impairment and lysosomal dysfunction [137] (Table 1).

The variability in findings from MA studies based on different models and strategies might reflect some previously established known problems with monitoring MA activity with in vitro and in vivo models [152]. Most studies revealed an alteration in MA, but the interpretation was sometimes made on the basis of data that were contradictory; for example, in some cases, elevated basal levels of LC3-II/p62 were attributed to MA blockage, and in other cases, MA blockade was attributed to decreased basal LC3-II levels, which corresponded to a lower number of autophagosomes.

Works based on depletion of GBA activity, such as studies using CBE, siRNA or GBA KO, seem to reveal a stronger effect on the MA pathway (see (Table 1); however, total loss of GCase activity did not recapitulate the PD-GBA context, in which most patients carry a heterozygous mutation and thus, maintain partial GBA activity. In this PD context with mutant GBA, the formation of autophagosomes and the autophagosome–lysosome fusion was apparently largely preserved.

In many of these studies, a more in-depth analysis of MA was performed via autophagic flux assays. As with the aforementioned work, despite a general interpretation claiming impairment to MA flux, in almost all studies, autophagosomes had clearly formed and later fused with lysosomes, indicating that MA was active but possibly showed lower effectiveness. Certainly, complementary assays such as autophagic cargo flux assays [153] or long-term proteolysis assays [154] can provide supplementary information to analyze the MA function in the presence of GBA mutations or GCase activity loss.

#### 6.2.5. Other Pathways Related to Macroautophagy Dysfunction in PD-GBA

Another characteristic of PD-GBA is altered mitochondrial function. Alterations in the respiratory capacity, ATP production and fusion/fission mechanism of mitochondria seem to be consistent in the context of GBA deficiency [82,131,134,137,155,156,157,158]. This mitochondrial dysfunction has been suggested to be a consequence of mitophagy impairment since autophagy–lysosome function has been shown to be dysregulated in GBA-related models. Whether the mitochondrial dysfunction observed in PD-GBA models and patient-derived tissues was caused directly by mitophagy impairment or by another mechanism, however, remains to be elucidated. To date, few works have directly detected impairment to mitophagy flux in cell models [134] or in postmortem brain tissue from PD patients carrying heterozygous GBA mutations [137]. Notably, several works have shown consistent mitochondrial dysfunction but not clear MA failure, suggesting that non-mitophagy mechanisms must be involved in the generation of mitochondrial dysfunction caused by GBA mutations.

Another interesting research topic emerging from studies on GBA-related MA impairment involves the deregulation of the exocytosis mechanism. Increasing evidence has pointed towards a key role for the cell-to-cell transmission of α-synuclein in promoting PD pathology in the brain, and importantly, in the PD-GBA context, GCase deficiency has been shown to increase the propagation of α-synuclein accumulation through cell-to-cell transmission [140,159,160,161]. The impairment of the autophagy–lysosome system can result in an increase in the number of released exosomes [160,162,163], and indeed, pharmacological inhibition of autophagic-lysosomal machinery with bafilomycin A_1_ has been shown to increase the level of extracellular α-synuclein and the association of α-synuclein with extracellular vesicles (EVs) derived from neuronal cells [164]. The extracellular release of α-synuclein has been linked to ceramide metabolism and to GBA mutant cell lines, with MA impairment as the leading factor triggering this effect [143,159,165].

## 7. Chaperone-Mediated Autophagy Impairment Related to GBA Dysfunction

### 7.1. CMA Pathway

CMA is an autophagic mechanism by which selective long-lived cytosolic proteins are recognized and internalized into lysosomes for recycling [166]. Only selective proteins carrying specific KFERQ-like targeting undergo degradation via CMA [167]. This degradative process is crucial for cells, since approximately 30% of cytosolic proteins in the mammalian proteome carry a CMA-targeting motif and are candidates for selective degradation through this pathway. Additionally, the targeting motif can be generated after posttranslational modification, increasing the number of potential CMA substrates [168]. 

CMA is a multistep process that is initiated when the cytosolic hsc70 chaperone, together with other cochaperones, recognizes the KFERQ-like motif in cytosolic proteins [166]. Upon recognition, the substrate protein is transported to the lysosome, where Hsc70 interacts with monomeric LAMP-2A, one of three isoforms of the LAMP-2 gene. The interaction drives the multimerization of LAMP-2A to form the CMA translocation complex and facilitates the translocation of the substrate across the lysosomal membrane, which is facilitated by lysosomal hsc70. Once in the lysosome, the targeted protein is degraded by lysosomal proteases [169].

CMA activity depends on the translocation complex, and accordingly, LAMP-2A is the rate-limiting factor in this selective pathway. Therefore, changes in the rates and dynamics of LAMP-2A at the lysosomal membrane modulate CMA activity. LAMP-2A levels at the lysosomal membrane are regulated mostly via its degradation, which is tightly regulated and involves sequential cleavage by cathepsin A and a membrane-associated metalloprotease that only takes place when LAMP-2A is present inside lipid microdomains. In contrast, outside lipid microdomains, LAMP-2A interacts with the substrate–chaperone complex and assembles the translocation complex to complete the degradation of the substrate [99,170].

Alterations in lipid levels and lysosomal membrane composition can affect the number of lipid microdomains, the level and stability of LAMP-2A and ultimately CMA activity [73,98,99].

### 7.2. CMA in Parkinson’s Disease: CMA-Dependent Degradation of α-Synuclein

PD has been linked to altered CMA machinery in multiple studies. Specifically, a reduction in the CMA-related proteins LAMP-2A and hsc70 has been observed in postmortem SN samples of PD brains, in contrast to the levels in healthy brains [171,172]. Decreased LAMP-2 gene expression has been found in peripheral leukocytes from sporadic PD patients, and in these samples, LC3 gene and protein level expression was increased, consistent with impaired CMA and upregulated MA [136].

LAMP-2A gene expression and protein levels have also been found to be decreased in other PD brain samples. In this study, the decrease was selective, as indicated because the levels of the other isoforms, LAMP-2B and LAMP-2C, were not affected and because the change was accompanied by a decrease in the level of the chaperone hsc70 and an increase in the level of α-synuclein, revealing CMA impairment associated with PD [172].

Additional studies in which PD-derived samples, such as lymphomonocytes and PBMCs, were analyzed, showed changes in CMA-associated gene expression and proteins [173,174,175].

To explain the connection between PD and CMA, different studies have shown that a decrease in α-synuclein turnover mediated by CMA might be a mechanism underlying the accumulation of α-synuclein in PD. 

In 2004, α-synuclein was described as a CMA substrate [176]. Wild-type α-synuclein carrying the KFERQ-like motif can be recognized by hsc70 and can bind LAMP-2A at the lysosomal membrane, leading to α-synuclein degradation via CMA machinery (Figure 1). However, although the pathogenic α-synuclein A30P and A53T mutants as well as posttranslationally modified α-synuclein bound to LAMP-2A, they were not translocated into the lysosome for degradation. Additionally, although both mutant α-synuclein and dopamine-modified α-synuclein species were able to bind LAMP-2A at the translocation complex, these α-synuclein species are not able to be translocated into the lysosomes and remain stacked, blocking CMA machinery and avoiding the degradation of other CMA substrates [176,177]. Overall, a decrease in the degradation of these α-synuclein species favored an increase in α-synuclein forms in the cytosol and promoted the formation of oligomeric protofibrillary intermediates, which usually form insoluble α-synuclein fibrils. Consistent with these findings, different studies have confirmed a key role for CMA in α-synuclein turnover in cell and animal models [178,179,180,181].

Confirming the link between CMA activity and α-synuclein turnover, the activation of CMA has been shown to be a new strategy to promote the rescue of normal levels of α-synuclein. LAMP-2A overexpression protected against α-synuclein-induced toxicity in different in vitro and in vivo models, including dopaminergic cells, primary cortical neurons and nigral dopaminergic neurons in the rat brain [73,179,182].

### 7.3. CMA in Parkinson’s Disease Associated with GBA

Recently, a link between the loss of GBA function and CMA activity has been proposed to explain the increase in α-synuclein levels associated with the loss of GBA activity.

Initially, some authors proposed that GBA loss of function can affect lysosomal function and that this lysosomal impairment can affect CMA activity, leading to a decrease in α-synuclein turnover; however, the molecular mechanisms underlying this link was not shown [89,127]. Recently, other works have proposed new mechanisms to explain the link between GBA mutations, CMA activity and α-synuclein accumulation in the context of PD associated with GBA. In vitro dopaminergic cell lines expressing mutant or with GBA knocked out showed extensive lysosomal dysfunction that favored the enrichment of sphingolipids and cholesterol organized as lipid microdomains in the lysosomal membrane [73]. Under these conditions, monomeric LAMP-2A was recruited to the lipid-enriched microdomains, where it underwent degradation by cathepsin A [99], preventing the assembly of the CMA-translocation complex and abrogating CMA activity. As previously shown in other models, this inhibition of CMA activity promoted the abnormal accumulation of α-synuclein and is among the alternative and convergent pathways that contribute to altering α-synuclein turnover and metabolism [73] (Figure 1). 

As a complementary hypothesis compatible with the aforementioned hypothesis, other authors have described a new mechanism of CMA inhibition based on the gain-of-function of a mutant GBA protein [139]. In this model, the GBA protein is retrotranslocated from the ER to the cytosol and then degraded via CMA. However, mutant GBA, in contrast to wild-type GBA, binds to CMA machinery but is not translocated into the lysosome, thereby blocking CMA machinery; this mechanism is analogous to other previously observed mechanisms with mutant α-synuclein, mutant LRRK2 and UCHL-1 [176,183,184].

## 8. Conclusions

In conclusion, several mechanisms initiated by mutations in the GBA enzyme can promote α-synuclein accumulation and aggregation. The direct role of abnormal levels of sphingolipids and cholesterol can directly favor an increase of α-synuclein neurotoxic species. In addition, lysosomal dysfunction clearly contributes to a decrease in the efficacy of lysosomal degradation during the final phase of the autophagy pathways. In addition, MA and CMA pathways can also be directly affected by a gain-of-function mutant GBA that induces toxicity and by alterations in lysosomal lipids. Therefore, the lysosomal-dependent degradation of α-synuclein is compromised, and α-synuclein accumulation over the pathogenic threshold is favored.

However, the link between GBA and PD is not limited to α-synuclein metabolism. Numerous pathological processes, in addition to α-synuclein levels, conformation or localization, underlie PD-GBA etiology. Wide-ranging lysosomal dysfunction not only affects all autophagic pathways and cellular proteostasis but also exerts an important impact in other systems where lysosomal function plays a key role, such as the endosomal and exocytic pathways. Finally, many other lysosome-independent mechanisms, not evaluated in detail in this review, such as ER stress caused by mutant GBA, mitochondrial dysfunction, oxidative stress, dysregulated calcium metabolism and many other converging cellular mechanisms, all contribute to the pathogenesis of PD and can be influenced and amplified by GBA mutations.

## Figures and Tables

**Table 1:** Macroautophagy alteration in vitro and in vivo GBA models.

Model		Sample	MA markers	Interpretation	Reference
**CBE**	**In vitro**	SH-SY5Y cells and rat cortical neurons	= LC3-II = Syn	No alterations in MA nor CMA	[126]
		SH-SY5Y cells	↑ LC3-II, p62, Syn ↓ mTORC1	defect in the “autolysosome reformation machinery”	[127]
	**In vivo**	Mouse	↑ LC3-II, p62 (SNpc) ↑ proteinase-K resistant agg-Syn	Impaired MA flux	[128]
**shRNA**	**In vitro**	Human neuroglioma (H4) + shRNA GBA	↑ LC3-II and LAMPs	GCase depletion causes a decline in lysosomal proteolysis that affects syn homeostasis	[129]
		SK-N-SH and rat cortical neuron	↓ LC3-II ↓ autophagic flux ↑ α-Syn	Autophagy pathway is severely compromised inhibiting MA induction via-mTORC1	[130]
		SH-SY5Y cells	↑ LC3-II, p62	Impairment of the lysosomal function	[127]
**KO**	**In vitro**	Mixed cultures of cortical neurons and astrocytes from GBA-/- mice	↓ LC3-II, Atg5-12 ↑ p62, ↑ α-Syn aggregated	Autophagy pathway is severely compromised	[131]
		HEK293 GBA-KO	↑ MA markers (LAMP-2, LC3B, p62, Rab7)	Attributed to the accumulation of toxic GlcSph	[81]
		Immortalized neurons	↑ LC3-II hyperactivated mTORC1	Altered lysosomes and autophagy, decrease	[82]
		BE(2)M17 GBA-KO cells	= LC3-II, p62 ↓ Flux	MA is affected very lightly	[73]
	**In vivo**	*Drosophila*	↑ LC3-II, p62 blocked MA flux	Failure in MA and lysosomal dysfunction	[132]
**Mutant GBA**	**In vitro**	Fibroblasts PD-GBA patients	= LC3-II	Lysosomal dysfunction but no MA alterations	[133]
		Fibroblasts from GD and GBA-PD patients	↑ LC3-II MA flux blockade	Impaired autophagic flux	[134]
		Fibroblasts from PD-GBA patients	↑ LC3-II, p62, α-Syn ↓ mTORC1	Defect in the “autolysosome reformation machinery”	[127]
		Fibroblasts from N370/WT patient	↑ LC3-II, p62 small effect on MA flux	Autophagy impairment cholesterol accumulation	[89]
		Fibroblasts iPD and PD-GBA patients	impaired autophagic flux	Impaired autophagic flux in PD-GBA	[135]
		PBMCs from PD patients	↑ LC3-II (mRNA and protein)	MA induction probably as a compensatory mechanism of CMA impairment	[136]
		SH-SY5Y + GBA L444P	↓ Mitophagy MA flux is working but in L444P is lower.	Mitophagy dysfunction and autophagy problesms	[137]
		Neural crest stem cell derived dopaminergic neurons	↓ Cat D: = Cat B = LAMP-1	GBA1 mutations lead to a lower level of cathepsin D protein and activity	[138]
		BE(2)M17 GBA-N370S and L444P cells	= LC3-II, p62 ↓ Flux	MA flux is slighted affected, MA induction activated to compensate lysosomal dysfunction.	[73]
		SH-SY5Y + GBA-WT/N370S, L444P, D409H and mouse primary neurons + WT/N370S GBA	↑ MA flux	MA is OK and over activated to compensate CMA dysfunction.	[139]
		iPSC-DA	↑ p62	Lysosomal dysfunction	[140]
		iPSC-DA from GBA-PD patients (N370S/WT and L444P/WT)	↑ LC3-II ↓ Flux	Autophagic and lysosomal defects.	[141]
		iPSC-DA from neuronopathic GD	↑ LC3-II ↓ Flux ↓ TFEB expression	Lysosomal dysfunction	[142]
		iPSC-DA N370S	↑ LC3-II, beclin 1, p62	Autophagic/lysosomal perturbations.	[143]
	**In vivo**	Mouse D409V knock-in	= LC3, p62, LAMP-2 ↓ Beclin	No differences in lysosomal and MA markers except for beclin (impairment in initiation of autophagosome).	[144]
		GBA L444P knockin mice	↑ basal LC3-II, p62 Other markers: mTOR, beclin.	impaired basal autophagy and lysosomal degradation MA Flux blocked. mitophagy impairment	[137]
		Mouse D409V knock-in	= LAMP-2	No changes in autophagy-lysosomal system	[145]
		Mouse D409V (+ ATP13A2)	= LAMP-2	No changes in autophagy-lysosomal system	[146]

## References

[1] Levine B., Klionsky D.J. (2017). Autophagy Wins the 2016 Nobel Prize in Physiology or Medicine: Breakthroughs in Baker’s Yeast Fuel Advances in Biomedical Research. Proc. Natl. Acad. Sci. USA.

[2] Kaushik S., Tasset I., Arias E., Pampliega O., Wong E., Martinez-Vicente M., Cuervo A.M. (2021). Autophagy and the Hallmarks of Aging. Ageing Res. Rev..

[3] Klionsky D.J., Petroni G., Amaravadi R.K., Baehrecke E.H., Ballabio A., Boya P., Bravo-San Pedro J.M., Cadwell K., Cecconi F., Choi A.M.K. (2021). Autophagy in Major Human Diseases. EMBO J..

[4] Hara T., Nakamura K., Matsui M., Yamamoto A., Nakahara Y., Suzuki-Migishima R., Yokoyama M., Mishima K., Saito I., Okano H. (2006). Suppression of Basal Autophagy in Neural Cells Causes Neurodegenerative Disease in Mice. Nature.

[5] Komatsu M., Waguri S., Chiba T., Murata S., Iwata J.I., Tanida I., Ueno T., Koike M., Uchiyama Y., Kominami E. (2006). Loss of Autophagy in the Central Nervous System Causes Neurodegeneration in Mice. Nature.

[6] Ahmed I., Liang Y., Schools S., Dawson V.L., Dawson T.M., Savitt J.M. (2012). Development and Characterization of a New Parkinson’s Disease Model Resulting from Impaired Autophagy. J. Neurosci..

[7] Sato S., Uchihara T., Fukuda T., Noda S., Kondo H., Saiki S., Komatsu M., Uchiyama Y., Tanaka K., Hattori N. (2018). Loss of Autophagy in Dopaminergic Neurons Causes Lewy Pathology and Motor Dysfunction in Aged Mice. Sci. Rep..

[8] Bourdenx M., Martín-Segura A., Scrivo A., Rodriguez-Navarro J.A., Kaushik S., Tasset I., Diaz A., Storm N.J., Xin Q., Juste Y.R. (2021). Chaperone-Mediated Autophagy Prevents Collapse of the Neuronal Metastable Proteome. Cell.

[9] Martinez-Vicente M. (2015). Autophagy in Neurodegenerative Diseases: From Pathogenic Dysfunction to Therapeutic Modulation. Semin. Cell Dev. Biol..

[10] Stavoe A.K.H., Holzbaur E.L.F., Stavoe A.K.H., Holzbaur E.L.F., Holzbaur E.L.F. (2019). Neuronal Autophagy Declines Substantially with Age and Is Rescued by Overexpression of WIPI2 Overexpression of WIPI2. Autophagy.

[11] Poewe W., Seppi K., Tanner C.M., Halliday G.M., Brundin P., Volkmann J., Schrag A.-E., Lang A.E. (2017). Parkinson Disease. Nat. Rev. Dis. Prim..

[12] Tanner C.M. (1992). Epidemiology of Parkinson’s Disease. Neurol. Clin..

[13] Surmeier D.J., Obeso J.A., Halliday G.M. (2017). Selective Neuronal Vulnerability in Parkinson Disease. Nat. Rev. Neurosci..

[14] Braak H., Del Tredici K., Bratzke H., Hamm-Clement J., Sandmann-Keil D., Rüb U. (2002). Staging of the Intracerebral Inclusion Body Pathology Associated with Idiopathic Parkinson’s Disease (Preclinical and Clinical Stages). J. Neurol..

[15] Spillantini M.G., Schmidt M.L., Lee V.M., Trojanowski J.Q., Jakes R., Goedert M. (1997). Alpha-Synuclein in Lewy Bodies. Nature.

[16] Mahul-Mellier A.-L., Burtscher J., Maharjan N., Weerens L., Croisier M., Kuttler F., Leleu M., Knott G.W., Lashuel H.A. (2020). The Process of Lewy Body Formation, Rather than Simply α-Synuclein Fibrillization, Is One of the Major Drivers of Neurodegeneration. Proc. Natl. Acad. Sci. USA.

[17] Klein A.D., Mazzulli J.R. (2018). Is Parkinson’s Disease a Lysosomal Disorder?. Brain.

[18] Goedert M., Jakes R., Spillantini M.G. (2017). The Synucleinopathies: Twenty Years On. J. Parkinsons. Dis..

[19] Bandres-Ciga S., Diez-Fairen M., Kim J.J., Singleton A.B. (2020). Genetics of Parkinson’s Disease: An Introspection of Its Journey towards Precision Medicine. Neurobiol. Dis..

[20] Lesage S., Brice A. (2009). Parkinson’s Disease: From Monogenic Forms to Genetic Susceptibility Factors. Hum. Mol. Genet..

[21] Simón-Sánchez J., Schulte C., Bras J.M., Sharma M., Gibbs J.R., Berg D., Paisan-Ruiz C., Lichtner P., Scholz S.W., Hernandez D.G. (2009). Genome-Wide Association Study Reveals Genetic Risk Underlying Parkinson’s Disease. Nat. Genet..

[22] Chang D., Nalls M.A., Hallgrímsdóttir I.B., Hunkapiller J., van der Brug M., Cai F., Kerchner G.A., Ayalon G., Bingol B., Sheng M. (2017). A Meta-Analysis of Genome-Wide Association Studies Identifies 17 New Parkinson’s Disease Risk Loci. Nat. Genet..

[23] Nalls M.A., Blauwendraat C., Vallerga C.L., Heilbron K., Bandres-Ciga S., Chang D., Tan M., Kia D.A., Noyce A.J., Xue A. (2019). Identification of Novel Risk Loci, Causal Insights, and Heritable Risk for Parkinson’s Disease: A Meta-Analysis of Genome-Wide Association Studies. Lancet Neurol..

[24] Nalls M.A., Pankratz N., Lill C.M., Do C.B., Hernandez D.G., Saad M., DeStefano A.L., Kara E., Bras J., Sharma M. (2014). Large-Scale Meta-Analysis of Genome-Wide Association Data Identifies Six New Risk Loci for Parkinson’s Disease. Nat. Genet..

[25] Navarro-Romero A., Montpeyó M., Martinez-Vicente M. (2020). The Emerging Role of the Lysosome in Parkinson’s Disease. Cells.

[26] Sidransky E., Samaddar T., Tayebi N. (2009). Mutations in GBA Are Associated with Familial Parkinson Disease Susceptibility and Age at Onset. Neurology.

[27] Sidransky E., Nalls M.A.A., Aasly J.O.O., Aharon-Peretz J., Annesi G., Barbosa E.R.R., Bar-Shira A., Berg D., Bras J., Brice A. (2009). Multicenter Analysis of Glucocerebrosidase Mutations in Parkinson’s Disease. N. Engl. J. Med..

[28] McNeill A., Duran R., Hughes D.A., Mehta A., Schapira A.H.V. (2012). A Clinical and Family History Study of Parkinson’s Disease in Heterozygous Glucocerebrosidase Mutation Carriers. J. Neurol. Neurosurg. Psychiatry.

[29] Vieira S.R.L., Schapira A.H.V. (2022). Glucocerebrosidase Mutations and Parkinson Disease. J. Neural Transm..

[30] Smith L., Schapira A.H.V. (2022). V GBA Variants and Parkinson Disease: Mechanisms and Treatments. Cells.

[31] Schapira A.H. (2015). V Glucocerebrosidase and Parkinson Disease: Recent Advances. Mol. Cell. Neurosci..

[32] O’Regan G., Desouza R.M., Balestrino R., Schapira A.H., O’ Regan G., Balestrino R., Schapira A.H. (2017). Glucocerebrosidase Mutations in Parkinson Disease. J. Parkinsons. Dis..

[33] Höglinger G., Schulte C., Jost W.H., Storch A., Woitalla D., Krüger R., Falkenburger B., Brockmann K., Storch A., Woitalla D. (2022). GBA-Associated PD: Chances and Obstacles for Targeted Treatment Strategies. J. Neural Transm..

[34] Neumann J., Bras J., Deas E., O’sullivan S.S., Parkkinen L., Lachmann R.H., Li A., Holton J., Guerreiro R., Paudel R. (2009). Glucocerebrosidase Mutations in Clinical and Pathologically Proven Parkinson’s Disease. Brain.

[35] Gan-Or Z., Giladi N., Rozovski U., Shifrin C., Rosner S., Gurevich T., Bar-Shira A., Orr-Urtreger A. (2008). Genotype-Phenotype Correlations between GBA Mutations and Parkinson Disease Risk and Onset. Neurology.

[36] Cilia R., Tunesi S., Marotta G., Cereda E., Siri C., Tesei S., Zecchinelli A.L., Canesi M., Mariani C.B., Meucci N. (2016). Survival and Dementia in GBA-Associated Parkinson’s Disease: The Mutation Matters. Ann. Neurol..

[37] Liu G., Boot B., Locascio J.J., Jansen I.E., Winder-Rhodes S., Eberly S., Elbaz A., Brice A., Ravina B., van Hilten J.J. (2016). Specifically Neuropathic Gaucher’s Mutations Accelerate Cognitive Decline in Parkinson’s. Ann. Neurol..

[38] Thaler A., Bregman N., Gurevich T., Shiner T., Dror Y., Zmira O., Gan-Or Z., Bar-Shira A., Gana-Weisz M., Orr-Urtreger A. (2018). Parkinson’s Disease Phenotype Is Influenced by the Severity of the Mutations in the GBA Gene. Parkinsonism Relat. Disord..

[39] Anheim M., Elbaz A., Lesage S., Durr A., Condroyer C., Viallet F., Pollak P., Bonaïti B., Bonaïti-Pellié C., Brice A. (2012). Penetrance of Parkinson Disease in Glucocerebrosidase Gene Mutation Carriers. Neurology.

[40] Lerche S., Schulte C., Wurster I., Machetanz G., Roeben B., Zimmermann M., Deuschle C., Hauser A.-K.K., Böhringer J., Krägeloh-Mann I. (2021). The Mutation Matters: CSF Profiles of GCase, Sphingolipids, α-Synuclein in PDGBA. Mov. Disord..

[41] Lerche S., Wurster I., Roeben B., Zimmermann M., Riebenbauer B., Deuschle C., Hauser A., Schulte C., Berg D., Maetzler W. (2020). Parkinson’s Disease: Glucocerebrosidase 1 Mutation Severity Is Associated with CSF Alpha-Synuclein Pro Fi Les. Mov. Disord..

[42] Nalls M.A., Duran R., Lopez G., Kurzawa-Akanbi M., McKeith I.G., Chinnery P.F., Morris C.M., Theuns J., Crosiers D., Cras P. (2013). A Multicenter Study of Glucocerebrosidase Mutations in Dementia with Lewy Bodies. JAMA Neurol..

[43] Greuel A., Trezzi J.P., Glaab E., Ruppert M.C., Maier F., Jäger C., Hodak Z., Lohmann K., Ma Y., Eidelberg D. (2020). GBA Variants in Parkinson’s Disease: Clinical, Metabolomic, and Multimodal Neuroimaging Phenotypes. Mov. Disord..

[44] Krohn L., Ruskey J.A., Rudakou U., Leveille E., Asayesh F., Hu M.T.M., Arnulf I., Dauvilliers Y., Högl B., Stefani A. (2020). GBA Variants in REM Sleep Behavior Disorder: A Multicenter Study. Neurology.

[45] Outeiro T.F., Koss D.J., Erskine D., Walker L., Kurzawa-Akanbi M., Burn D., Donaghy P., Morris C., Taylor J.-P., Thomas A. (2019). Dementia with Lewy Bodies: An Update and Outlook. Mol. Neurodegener..

[46] Sklerov M., Kang U.J., Liong C., Clark L., Marder K., Pauciulo M., Nichols W.C., Chung W.K., Honig L.S., Cortes E. (2017). Frequency of GBA Variants in Autopsy-Proven Multiple System Atrophy. Mov. Disord. Clin. Pract..

[47] Miyoshi K., Hagita H., Horiguchi T., Tanimura A., Noma T. (2022). Redefining GBA Gene Structure Unveils the Ability of Cap-Independent, IRES-Dependent Gene Regulation. Commun. Biol..

[48] Horowitz M., Wilder S., Horowitz Z., Reiner O., Gelbart T., Beutler E. (1989). The Human Glucocerebrosidase Gene and Pseudogene: Structure and Evolution. Genomics.

[49] Straniero L., Rimoldi V., Samarani M., Goldwurm S., Di Fonzo A., Krüger R., Deleidi M., Aureli M., Soldà G., Duga S. (2017). The GBAP1 Pseudogene Acts as a CeRNA for the Glucocerebrosidase Gene GBA by Sponging MiR-22-3p. Sci. Rep..

[50] Brady R.O., Kanfer J., Shapiro D. (1965). The Metabolism of Glucocerebrosides. I. Purification and Properties of a Glucocerebroside-cleaving Enzyme From Spleen Tissue. J. Biol. Chem..

[51] Dvir H., Harel M., McCarthy A.A., Toker L., Silman I., Futerman A.H., Sussman J.L. (2003). X-ray Structure of Human Acid-β-glucosidase, the Defective Enzyme in Gaucher Disease. EMBO Rep..

[52] Smith L., Mullin S., Schapira A.H.V. (2017). Insights into the Structural Biology of Gaucher Disease. Exp. Neurol..

[53] Reczek D., Schwake M., Schröder J., Hughes H., Blanz J., Jin X., Brondyk W., Van Patten S., Edmunds T., Saftig P. (2007). LIMP-2 Is a Receptor for Lysosomal Mannose-6-Phosphate-Independent Targeting of β-Glucocerebrosidase. Cell.

[54] Malini E., Zampieri S., Deganuto M., Romanello M., Sechi A., Bembi B., Dardis A. (2015). Role of LIMP-2 in the Intracellular Trafficking of β-Glucosidase in Different Human Cellular Models. FASEB J..

[55] Blanz J., Zunke F., Markmann S., Damme M., Braulke T., Saftig P., Schwake M. (2015). Mannose 6-Phosphate-Independent Lysosomal Sorting of LIMP-2. Traffic.

[56] Zachos C., Blanz J., Saftig P., Schwake M. (2012). A Critical Histidine Residue Within LIMP-2 Mediates PH Sensitive Binding to Its Ligand β-Glucocerebrosidase. Traffic.

[57] Liou B., Haffey W.D., Greis K.D., Grabowski G.A. (2014). The LIMP-2/SCARB2 Binding Motif on Acid β-Glucosidase. J. Biol. Chem..

[58] Zunke F., Andresen L., Wesseler S., Groth J., Arnold P., Rothaug M., Mazzulli J.R., Krainc D., Blanz J., Saftig P. (2016). Characterization of the Complex Formed by β-Glucocerebrosidase and the Lysosomal Integral Membrane Protein Type-2. Proc. Natl. Acad. Sci. USA.

[59] Abdul-Hammed M., Breiden B., Schwarzmann G., Sandhoff K. (2017). Lipids Regulate the Hydrolysis of Membrane Bound Glucosylceramide by Lysosomal β-Glucocerebrosidase. J. Lipid Res..

[60] Atrian S., López-Viñas E., Gómez-Puertas P., Chabás A., Vilageliu L., Grinberg D. (2008). An Evolutionary and Structure-based Docking Model for Glucocerebrosidase–saposin C and Glucocerebrosidase–substrate Interactions—Relevance for Gaucher Disease. Proteins Struct. Funct. Bioinforma..

[61] Aerts J.M.F.G., Sa Miranda M.C., Brouwer-Kelder E.M., Van Weely S., Barranger J.A., Tager J.M. (1990). Conditions Affecting the Activity of Glucocerebrosidase Purified from Spleens of Control Subjects and Patients with Type 1 Gaucher Disease. Biochim. Biophys. Acta.

[62] Wilkening G., Linke T., Sandhoff K. (1998). Lysosomal Degradation on Vesicular Membrane Surfaces. J. Biol. Chem..

[63] Salvioli R., Tatti M., Scarpa S., Moavero S.M., Ciaffoni F., Felicetti F., Kaneski C.R., Brady R.O., Vaccaro A.M. (2005). The N370S (Asn370→Ser) Mutation Affects the Capacity of Glucosylceramidase to Interact with Anionic Phospholipid-Containing Membranes and Saposin C. Biochem. J..

[64] Futerman A.H., Platt F.M. (2017). The Metabolism of Glucocerebrosides—From 1965 to the Present. Mol. Genet. Metab..

[65] Hein L.K., Rozaklis T., Adams M.K., Hopwood J.J., Karageorgos L. (2017). Lipid Composition of Microdomains Is Altered in Neuronopathic Gaucher Disease Sheep Brain and Spleen. Mol. Genet. Metab..

[66] Karageorgos L., Hein L., Rozaklis T., Adams M., Duplock S., Snel M., Hemsley K., Kuchel T., Smith N., Hopwood J.J. (2016). Glycosphingolipid Analysis in a Naturally Occurring Ovine Model of Acute Neuronopathic Gaucher Disease. Neurobiol. Dis..

[67] Ghauharali-van der Vlugt K., Langeveld M., Poppema A., Kuiper S., Hollak C.E.M., Aerts J.M., Groener J.E.M. (2008). Prominent Increase in Plasma Ganglioside GM3 Is Associated with Clinical Manifestations of Type I Gaucher Disease. Clin. Chim. Acta.

[68] Rolfs A., Giese A.K., Grittner U., Mascher D., Elstein D., Zimran A., Böttcher T., Lukas J., Hübner R., Gölnitz U. (2013). Glucosylsphingosine Is a Highly Sensitive and Specific Biomarker for Primary Diagnostic and Follow-up Monitoring in Gaucher Disease in a Non-Jewish, Caucasian Cohort of Gaucher Disease Patients. PLoS ONE.

[69] Murugesan V., Chuang W.L., Liu J., Lischuk A., Kacena K., Lin H., Pastores G.M., Yang R., Keutzer J., Zhang K. (2016). Glucosylsphingosine Is a Key Biomarker of Gaucher Disease. Am. J. Hematol..

[70] Dekker N., Van Dussen L., Hollak C.E.M., Overkleeft H., Scheij S., Ghauharali K., Van Breemen M.J., Ferraz M.J., Groener J.E.M., Maas M. (2011). Elevated Plasma Glucosylsphingosine in Gaucher Disease: Relation to Phenotype, Storage Cell Markers, and Therapeutic Response. Blood.

[71] Muñoz S.S., Petersen D., Marlet F.R., Kücükköse E., Galvagnion C. (2021). The Interplay between Glucocerebrosidase, α-Synuclein and Lipids in Human Models of Parkinson’s Disease. Biophys. Chem..

[72] Rocha E.M., Smith G.A., Park E., Cao H., Brown E., Hallett P., Isacson O. (2015). Progressive Decline of Glucocerebrosidase in Aging and Parkinson’s Disease. Ann. Clin. Transl. Neurol..

[73] Navarro-Romero A., Fernandez-Gonzalez I., Riera J., Montpeyo M., Albert-Bayo M., Lopez-Royo T., Castillo-Sanchez P., Carnicer-Caceres C., Arranz-Amo J.A., Castillo-Ribelles L. (2022). Lysosomal Lipid Alterations Caused by Glucocerebrosidase Deficiency Promote Lysosomal Dysfunction, Chaperone-Mediated-Autophagy Deficiency, and Alpha-Synuclein Pathology. npj Park. Dis..

[74] Srikanth M.P., Jones J.W., Kane M., Awad O., Park T.S., Zambidis E.T., Feldman R.A. (2021). Elevated Glucosylsphingosine in Gaucher Disease Induced Pluripotent Stem Cell Neurons Deregulates Lysosomal Compartment through Mammalian Target of Rapamycin Complex 1. Stem Cells Transl. Med..

[75] Yamaguchi Y., Sasagasako N., Goto I., Kobayashi T. (1994). The Synthetic Pathway for Glucosylsphingosine in Cultured Fibroblasts. J. Biochem..

[76] Schueler U.H., Kolter T., Kaneski C.R., Blusztajn J.K., Herkenham M., Sandhoff K., Brady R.O. (2003). Toxicity of Glucosylsphingosine (Glucopsychosine) to Cultured Neuronal Cells: A Model System for Assessing Neuronal Damage in Gaucher Disease Type 2 and 3. Neurobiol. Dis..

[77] Van Eijk M., Ferra M.J., Boot R.G., Aerts J.M.F.G. (2020). Lyso-Glycosphingolipids: Presence and Consequences. Essays Biochem..

[78] van der Poel S., Wolthoorn J., van den Heuvel D., Egmond M., Groux-Degroote S., Neumann S., Gerritsen H., van Meer G., Sprong H. (2011). Hyperacidification of Trans-Golgi Network and Endo/Lysosomes in Melanocytes by Glucosylceramide-Dependent V-ATPase Activity. Traffic.

[79] Sillence D.J. (2013). Glucosylceramide Modulates Endolysosomal PH in Gaucher Disease. Mol. Genet. Metab..

[80] Sillence D.J., Puri V., Marks D.L., Butters T.D., Dwek R.A., Pagano R.E., Platt F.M. (2002). Glucosylceramide Modulates Membrane Traffic along the Endocytic Pathway. J. Lipid Res..

[81] Kim M.J., Jeon S., Burbulla L.F., Krainc D. (2018). Acid Ceramidase Inhibition Ameliorates a Synuclein Accumulation upon Loss of GBA1 Function. Hum. Mol. Genet..

[82] Peng Y., Liou B., Lin Y., Fannin V., Zhang W., Feldman R.A., Setchell K.D.R.R., Grabowski G.A., Sun Y. (2021). Substrate Reduction Therapy Reverses Mitochondrial, MTOR, and Autophagy Alterations in a Cell Model of Gaucher Disease. Cells.

[83] Ballabio A., Bonifacino J.S. (2020). Lysosomes as Dynamic Regulators of Cell and Organismal Homeostasis. Nat. Rev. Mol. Cell Biol..

[84] Perera R.M., Zoncu R. (2016). The Lysosome as a Regulatory Hub. Annu. Rev. Cell Dev. Biol..

[85] Rabanal-Ruiz Y., Korolchuk V. (2018). MTORC1 and Nutrient Homeostasis: The Central Role of the Lysosome. Int. J. Mol. Sci..

[86] Saxton R.A., Sabatini D.M. (2017). MTOR Signaling in Growth, Metabolism, and Disease. Cell.

[87] Rabanal-Ruiz Y., Otten E.G., Korolchuk V.I. (2017). MTORC1 as the Main Gateway to Autophagy. Essays Biochem..

[88] Meng Y., Heybrock S., Neculai D., Saftig P. (2020). Cholesterol Handling in Lysosomes and Beyond. Trends Cell Biol..

[89] García-Sanz P., Orgaz L., Bueno-Gil G., Espadas I., Rodríguez-Traver E., Kulisevsky J., Gutierrez A., Dávila J.C., González-Polo R.A., Fuentes J.M. (2017). N370S -GBA1 Mutation Causes Lysosomal Cholesterol Accumulation in Parkinson’s Disease. Mov. Disord..

[90] Akiyama H., Kobayashi S., Hirabayashi Y., Murakami-Murofushi K. (2013). Cholesterol Glucosylation Is Catalyzed by Transglucosylation Reaction of β-Glucosidase 1. Biochem. Biophys. Res. Commun..

[91] Marques A.A.R.A., Mirzaian M., Akiyama H., Wisse P., Ferraz M.J., Gaspar P., Ghauharali-van der Vlugt K., Meijer R., Giraldo P., Alfonso P. (2016). Glucosylated Cholesterol in Mammalian Cells and Tissues: Formation and Degradation by Multiple Cellular β-Glucosidases. J. Lipid Res..

[92] Franco R., Sánchez-Arias J.A., Navarro G., Lanciego J.L. (2018). Glucocerebrosidase Mutations and Synucleinopathies. Potential Role of Sterylglucosides and Relevance of Studying both GBA1 and GBA2 Genes. Front. Neuroanat..

[93] Conrad K.S., Cheng T.-W., Ysselstein D., Heybrock S., Hoth L.R., Chrunyk B.A., am Ende C.W., Krainc D., Schwake M., Saftig P. (2017). Lysosomal Integral Membrane Protein-2 as a Phospholipid Receptor Revealed by Biophysical and Cellular Studies. Nat. Commun..

[94] Neculai D., Schwake M., Ravichandran M., Zunke F., Collins R.F., Peters J., Neculai M., Plumb J., Loppnau P., Pizarro J.C. (2013). Structure of LIMP-2 Provides Functional Insights with Implications for SR-BI and CD36. Nature.

[95] Heybrock S., Kanerva K., Meng Y., Ing C., Liang A., Xiong Z.-J.J., Weng X., Ah Kim Y., Collins R., Trimble W. (2019). Lysosomal Integral Membrane Protein-2 (LIMP-2/SCARB2) Is Involved in Lysosomal Cholesterol Export. Nat. Commun..

[96] van der Lienden M.J.C., Aten J., Marques A.R.A., Waas I.S.E., Larsen P.W.B., Claessen N., van der Wel N.N., Ottenhoff R., van Eijk M., Aerts J.M.F.G. (2021). GCase and LIMP2 Abnormalities in the Liver of Niemann Pick Type C Mice. Int. J. Mol. Sci..

[97] Castellano B.M., Thelen A.M., Moldavski O., Feltes M., van der Welle R.E.N., Mydock-McGrane L., Jiang X., van Eijkeren R.J., Davis O.B., Louie S.M. (2017). Lysosomal Cholesterol Activates MTORC1 via an SLC38A9–Niemann-Pick C1 Signaling Complex. Science.

[98] Rodriguez-Navarro J.A., Cuervo A.M. (2012). Dietary Lipids and Aging Compromise Chaperone-Mediated Autophagy by Similar Mechanisms. Autophagy.

[99] Kaushik S., Massey A.C., Cuervo A.M. (2006). Lysosome Membrane Lipid Microdomains: Novel Regulators of Chaperone-Mediated Autophagy. EMBO J..

[100] Villar-Piqué A., Lopes da Fonseca T., Outeiro T.F. (2016). Structure, Function and Toxicity of Alpha-Synuclein: The Bermuda Triangle in Synucleinopathies. J. Neurochem..

[101] Ulmer T.S., Bax A., Cole N.B., Nussbaum R.L. (2005). Structure and Dynamics of Micelle-Bound Human Alpha-Synuclein. J. Biol. Chem..

[102] Middleton E.R., Rhoades E. (2010). Effects of Curvature and Composition on α-Synuclein Binding to Lipid Vesicles. Biophys. J..

[103] Kamp F., Beyer K. (2006). Binding of α-Synuclein Affects the Lipid Packing in Bilayers of Small Vesicles. J. Biol. Chem..

[104] Lee Y.J., Wang S., Slone S.R., Yacoubian T.A., Witt S.N. (2011). Defects in Very Long Chain Fatty Acid Synthesis Enhance Alpha-Synuclein Toxicity in a Yeast Model of Parkinson’s Disease. PLoS ONE.

[105] Leftin A., Job C., Beyer K., Brown M.F. (2013). Solid-State 13C NMR Reveals Annealing of Raft-like Membranes Containing Cholesterol by the Intrinsically Disordered Protein α-Synuclein. J. Mol. Biol..

[106] Varkey J., Isas J.M., Mizuno N., Jensen M.B., Bhatia V.K., Jao C.C., Petrlova J., Voss J.C., Stamou D.G., Steven A.C. (2010). Membrane Curvature Induction and Tubulation Are Common Features of Synucleins and Apolipoproteins. J. Biol. Chem..

[107] Rocha S., Kumar R., Nordén B., Wittung-Stafshede P. (2021). Orientation of α-Synuclein at Negatively Charged Lipid Vesicles: Linear Dichroism Reveals Time-Dependent Changes in Helix Binding Mode. J. Am. Chem. Soc..

[108] Fantini J., Carlus D., Yahi N. (2011). The Fusogenic Tilted Peptide (67–78) of α-Synuclein Is a Cholesterol Binding Domain. Biochim. Biophys. Acta Biomembr..

[109] Varkey J., Mizuno N., Hegde B.G., Cheng N., Steven A.C., Langen R. (2013). α-Synuclein Oligomers with Broken Helical Conformation Form Lipoprotein Nanoparticles. J. Biol. Chem..

[110] Koob A.O., Paulino A.D., Masliah E. (2010). GFAP Reactivity, Apolipoprotein E Redistribution and Cholesterol Reduction in Human Astrocytes Treated with α-Synuclein. Neurosci. Lett..

[111] Ronzitti G., Bucci G., Emanuele M., Leo D., Sotnikova T.D., Mus L.V., Soubrane C.H., Dallas M.L., Thalhammer A., Cingolani L.A. (2014). Exogenous -Synuclein Decreases Raft Partitioning of Cav2.2 Channels Inducing Dopamine Release. J. Neurosci..

[112] Perissinotto F., Rondelli V., Parisse P., Tormena N., Zunino A., Almásy L., Merkel D.G., Bottyán L., Sajti S., Casalis L. (2019). GM1 Ganglioside Role in the Interaction of Alpha-Synuclein with Lipid Membranes: Morphology and Structure. Biophys. Chem..

[113] Gaspar R., Pallbo J., Weininger U., Linse S., Sparr E. (2018). Ganglioside Lipids Accelerate α-Synuclein Amyloid Formation. Biochim. Biophys. Acta Proteins Proteom..

[114] Martinez Z., Zhu M., Han S., Fink A.L. (2007). GM1 Specifically Interacts with α-Synuclein and Inhibits Fibrillation. Biochemistry.

[115] O’Leary E.I., Jiang Z., Strub M.P., Lee J.C. (2018). Effects of Phosphatidylcholine Membrane Fluidity on the Conformation and Aggregation of N-Terminally Acetylated α-Synuclein. J. Biol. Chem..

[116] Paul A., Jacoby G., Laor Bar-Yosef D., Beck R., Gazit E., Segal D. (2021). Glucosylceramide Associated with Gaucher Disease Forms Amyloid-like Twisted Ribbon Fibrils That Induce α-Synuclein Aggregation. ACS Nano.

[117] Taguchi Y.V., Liu J., Ruan J., Pacheco J., Zhang X., Abbasi J., Keutzer J., Mistry P.K., Chandra S.S. (2017). Glucosylsphingosine Promotes α-Synuclein Pathology in Mutant GBA-Associated Parkinson’s Disease. J. Neurosci..

[118] Yap T.L., Gruschus J.M., Velayati A., Westbroek W., Goldin E., Moaven N., Sidransky E., Lee J.C. (2011). Alpha-Synuclein Interacts with Glucocerebrosidase Providing a Molecular Link between Parkinson and Gaucher Diseases. J. Biol. Chem..

[119] Kaur U., Lee J.C. (2021). Membrane Interactions of α-Synuclein Probed by Neutrons and Photons. Acc. Chem. Res..

[120] Yap T.L., Jiang Z., Heinrich F., Gruschus J.M., Pfefferkorn C.M., Barros M., Curtis J.E., Sidransky E., Lee J.C. (2015). Structural Features of Membrane-Bound Glucocerebrosidase and α-Synuclein Probed by Neutron Reflectometry and Fluorescence Spectroscopy. J. Biol. Chem..

[121] Yap T.L., Velayati A., Sidransky E., Lee J.C. (2013). Membrane-Bound α-Synuclein Interacts with Glucocerebrosidase and Inhibits Enzyme Activity. Mol. Genet. Metab..

[122] Yap T.L., Gruschus J.M., Velayati A., Sidransky E., Lee J.C. (2013). Saposin C Protects Glucocerebrosidase against α-Synuclein Inhibition. Biochemistry.

[123] Mizushima N., Levine B. (2020). Autophagy in Human Diseases. N. Engl. J. Med..

[124] Lamark T., Johansen T. (2021). Mechanisms of Selective Autophagy. Annu. Rev. Cell Dev. Biol..

[125] Levine B., Kroemer G. (2019). Biological Functions of Autophagy Genes: A Disease Perspective. Cell.

[126] Dermentzaki G., Dimitriou E., Xilouri M., Michelakakis H., Stefanis L. (2013). Loss of β-Glucocerebrosidase Activity Does Not Affect Alpha-Synuclein Levels or Lysosomal Function in Neuronal Cells. PLoS ONE.

[127] Magalhaes J., Gegg M.E., Migdalska-Richards A., Doherty M.K., Whitfield P.D., Schapira A.H.V. (2016). Autophagic Lysosome Reformation Dysfunction in Glucocerebrosidase Deficient Cells: Relevance to Parkinson Disease. Hum. Mol. Genet..

[128] Rocha E.M., Smith G.A., Park E., Cao H., Brown E., Hayes M.A., Beagan J., McLean J.R., Izen S.C., Perez-Torres E. (2015). Glucocerebrosidase Gene Therapy Prevents α-Synucleinopathy of Midbrain Dopamine Neurons. Neurobiol. Dis..

[129] Mazzulli J.R., Xu Y.-H., Sun Y., Knight A.L., McLean P.J., Caldwell G.A., Sidransky E., Grabowski G.A., Krainc D. (2011). Gaucher Disease Glucocerebrosidase and α-Synuclein Form a Bidirectional Pathogenic Loop in Synucleinopathies. Cell.

[130] Du T.-T., Wang L., Duan C.-L., Lu L.-L., Zhang J.-L., Gao G., Qiu X.-B., Wang X.-M., Yang H. (2015). GBA Deficiency Promotes SNCA/α-Synuclein Accumulation through Autophagic Inhibition by Inactivated PPP2A. Autophagy.

[131] Osellame L.D., Rahim A.A., Hargreaves I.P., Gegg M.E., Richard-Londt A., Brandner S., Waddington S.N., Schapira A.H.V., Duchen M.R. (2013). Mitochondria and Quality Control Defects in a Mouse Model of Gaucher Disease--Links to Parkinson’s Disease. Cell Metab..

[132] Kinghorn K.J., Grönke S., Castillo-Quan J.I., Woodling N.S., Li L., Sirka E., Gegg M., Mills K., Hardy J., Bjedov I. (2016). A Drosophila Model of Neuronopathic Gaucher Disease Demonstrates Lysosomal-Autophagic Defects and Altered MTOR Signalling and Is Functionally Rescued by Rapamycin. J. Neurosci..

[133] McNeill A., Magalhaes J., Shen C., Chau K.-Y.Y., Hughes D., Mehta A., Foltynie T., Cooper J.M., Abramov A.Y., Gegg M. (2014). Ambroxol Improves Lysosomal Biochemistry in Glucocerebrosidase Mutation-Linked Parkinson Disease Cells. Brain.

[134] de la Mata M., Cotán D., Oropesa-Ávila M., Garrido-Maraver J., Cordero M.D., Villanueva Paz M., Delgado Pavón A., Alcocer-Gómez E., De Lavera I., Ybot-González P. (2015). Pharmacological Chaperones and Coenzyme Q10 Treatment Improves Mutant β-Glucocerebrosidase Activity and Mitochondrial Function in Neuronopathic Forms of Gaucher Disease. Sci. Rep..

[135] Collins L.M., Drouin-Ouellet J., Kuan W.-L., Cox T., Barker R.A. (2017). Dermal Fibroblasts from Patients with Parkinson’s Disease Have Normal GCase Activity and Autophagy Compared to Patients with PD and GBA Mutations. F1000Research.

[136] Wu G., Wang X., Feng X., Zhang A., Li J., Gu K., Huang J., Pang S., Dong H., Gao H. (2011). Altered Expression of Autophagic Genes in the Peripheral Leukocytes of Patients with Sporadic Parkinson’s Disease. Brain Res..

[137] Li H., Ham A., Ma T.C., Kuo S.-H.H., Kanter E., Kim D., Ko H.S., Quan Y., Sardi S.P., Li A. (2019). Mitochondrial Dysfunction and Mitophagy Defect Triggered by Heterozygous GBA Mutations. Autophagy.

[138] Yang S.Y., Gegg M., Chau D., Schapira A. (2020). Glucocerebrosidase Activity, Cathepsin D and Monomeric α-Synuclein Interactions in a Stem Cell Derived Neuronal Model of a PD Associated GBA1 Mutation. Neurobiol. Dis..

[139] Kuo S.H., Tasset I., Cheng M.M., Diaz A., Pan M.K., Lieberman O.J., Hutten S.J., Alcalay R.N., Kim S., Ximénez-Embún P. (2022). Mutant Glucocerebrosidase Impairs α-Synuclein Degradation by Blockade of Chaperone-Mediated Autophagy. Sci. Adv..

[140] Bae E.-J., Yang N.-Y., Song M., Lee C.S., Lee J.S., Jung B.C., Lee H.-J., Kim S., Masliah E., Sardi S.P. (2014). Glucocerebrosidase Depletion Enhances Cell-to-Cell Transmission of α-Synuclein. Nat. Commun..

[141] Schöndorf D.C., Aureli M., McAllister F.E., Hindley C.J., Mayer F., Schmid B., Sardi S.P., Valsecchi M., Hoffmann S., Schwarz L.K. (2014). IPSC-Derived Neurons from GBA1-Associated Parkinson’s Disease Patients Show Autophagic Defects and Impaired Calcium Homeostasis. Nat. Commun..

[142] Awad O., Sarkar C., Panicker L.M., Miller D., Zeng X., Sgambato J.A., Feldman R.A. (2015). Altered TFEB-Mediated Lysosomal Biogenesis in Gaucher Disease IPSC-Derived Neuronal Cells. Hum. Mol. Genet..

[143] Fernandes H.J.R.R., Hartfield E.M., Christian H.C., Emmanoulidou E., Zheng Y., Booth H., Bogetofte H., Lang C., Ryan B.J., Sardi S.P. (2016). ER Stress and Autophagic Perturbations Lead to Elevated Extracellular α-Synuclein in GBA-N370S Parkinson’s IPSC-Derived Dopamine Neurons. Stem Cell Rep..

[144] Sardi S.P., Clarke J., Kinnecom C., Tamsett T.J., Li L., Stanek L.M., Passini M.A., Grabowski G.A., Schlossmacher M.G., Sidman R.L. (2011). CNS Expression of Glucocerebrosidase Corrects Alpha-Synuclein Pathology and Memory in a Mouse Model of Gaucher-Related Synucleinopathy. Proc. Natl. Acad. Sci. USA.

[145] Polinski N.K., Martinez T.N., Gorodinsky A., Gareus R., Sasner M., Herberth M., Switzer R., Ahmad S.O., Cosden M., Kandebo M. (2021). Decreased Glucocerebrosidase Activity and Substrate Accumulation of Glycosphingolipids in a Novel GBA1 D409V Knock-in Mouse Model. PLoS ONE.

[146] Johnson M.E., Bergkvist L., Stetzik L., Steiner J.A., Meyerdirk L., Schulz E., Wolfrum E., Luk K.C., Wesson D.W., Krainc D. (2021). Heterozygous GBA D490V and ATP13a2 Mutations Do Not Exacerbate Pathological α-Synuclein Spread in the Prodromal Preformed Fibrils Model in Young Mice. Neurobiol. Dis..

[147] Aerts J.M.F.G., Hollak C.E.M., Boot R.G., Groener J.E.M., Maas M. (2006). Substrate Reduction Therapy of Glycosphingolipid Storage Disorders. J. Inherit. Metab. Dis..

[148] Stirnemann J., Belmatoug N., Camou F., Serratrice C., Froissart R., Caillaud C., Levade T., Astudillo L., Serratrice J., Brassier A. (2017). A Review of Gaucher Disease Pathophysiology, Clinical Presentation and Treatments. Int. J. Mol. Sci..

[149] Smith L.J., Bolsinger M.M., Chau K.-Y., Gegg M.E., Schapira A.H. (2022). V The GBA Variant E326K Is Associated with Alpha-Synuclein Aggregation and Lipid Droplet Accumulation in Human Cell Lines. Hum. Mol. Genet..

[150] Jacobson M.A., Lipinski M.M., Feldman R.A. (2019). MTOR Hyperactivity Mediates Lysosomal Dysfunction in Gaucher’s Disease IPSC- Neuronal Cells. Dis. Model. Mech..

[151] Johri A., Chandra A., Beal M.F., Flint Beal M. (2013). PGC-1α, Mitochondrial Dysfunction, and Huntington’s Disease. Free Radic. Biol. Med..

[152] Klionsky D.J., Abdel-Aziz A.K., Abdelfatah S., Abdellatif M., Abdoli A., Abel S., Abeliovich H., Abildgaard M.H., Abudu Y.P., Acevedo-Arozena A. (2021). Guidelines for the Use and Interpretation of Assays for Monitoring Autophagy (4th Edition). Autophagy.

[153] Engedal N., Sønstevold T., Beese C.J., Selladurai S., Melcher T., Simensen J.E., Frankel L.B., Urbanucci A., Torgersen M.L. (2022). Measuring Autophagic Cargo Flux with Keima-Based Probes. Methods Mol. Biol..

[154] Luhr M., Sætre F., Engedal N. (2018). The Long-Lived Protein Degradation Assay: An Efficient Method for Quantitative Determination of the Autophagic Flux of Endogenous Proteins in Adherent Cell Lines. Bio-Protocol.

[155] Ivanova M.M., Changsila E., Iaonou C., Goker-Alpan O. (2019). Impaired Autophagic and Mitochondrial Functions Are Partially Restored by ERT in Gaucher and Fabry Diseases. PLoS ONE.

[156] Cleeter M.W.J., Chau K.-Y., Gluck C., Mehta A., Hughes D.A., Duchen M., Wood N.W., Hardy J., Mark Cooper J., Schapira A.H. (2013). Glucocerebrosidase Inhibition Causes Mitochondrial Dysfunction and Free Radical Damage. Neurochem. Int..

[157] Xu Y., Xu K., Sun Y., Liou B., Quinn B., Li R. (2014). Multiple Pathogenic Proteins Implicated in Neuronopathic Gaucher Disease Mice. Hum. Mol. Genet..

[158] Yun S.P., Kim D., Kim S., Kim S., Karuppagounder S.S., Kwon S.H., Lee S., Kam T.I., Lee S., Ham S. (2018). α-Synuclein Accumulation and GBA Deficiency Due to L444P GBA Mutation Contributes to MPTP-Induced Parkinsonism. Mol. Neurodegener..

[159] Gegg M.E., Verona G., Schapira A.H.V. (2020). Glucocerebrosidase Deficiency Promotes Release of α-Synuclein Fibrils from Cultured Neurons. Hum. Mol. Genet..

[160] Alvarez-Erviti L., Seow Y., Schapira A.H., Gardiner C., Sargent I.L., Wood M.J.A., Cooper J.M. (2011). Lysosomal Dysfunction Increases Exosome-Mediated Alpha-Synuclein Release and Transmission. Neurobiol. Dis..

[161] Cerri S., Ghezzi C., Ongari G., Croce S., Avenali M., Zangaglia R., Di Monte D.A., Valente E.M., Blandini F. (2021). GBA Mutations Influence the Release and Pathological Effects of Small Extracellular Vesicles from Fibroblasts of Patients with Parkinson’s Disease. Int. J. Mol. Sci..

[162] Fussi N., Höllerhage M., Chakroun T., Nykänen N.-P.P., Rösler T.W., Koeglsperger T., Wurst W., Behrends C., Höglinger G.U. (2018). Exosomal Secretion of α-Synuclein as Protective Mechanism after Upstream Blockage of Macroautophagy. Cell Death Dis..

[163] Poehler A.-M., Xiang W., Spitzer P., May V.E.L., Meixner H., Rockenstein E., Chutna O., Outeiro T.F., Winkler J., Masliah E. (2014). Autophagy Modulates SNCA/α-Synuclein Release, Thereby Generating a Hostile Microenvironment. Autophagy.

[164] Sagini K., Buratta S., Delo F., Pellegrino R.M., Giovagnoli S., Urbanelli L., Emiliani C. (2021). Drug-Induced Lysosomal Impairment Is Associated with the Release of Extracellular Vesicles Carrying Autophagy Markers. Int. J. Mol. Sci..

[165] Thomas R.E., Vincow E.S., Merrihew G.E., MacCoss M.J., Davis M.Y., Pallanck L.J. (2018). Glucocerebrosidase Deficiency Promotes Protein Aggregation through Dysregulation of Extracellular Vesicles. PLoS Genet..

[166] Cuervo A.M., Dice J.F. (1996). A Receptor for the Selective Uptake and Degradation of Proteins by Lysosomes. Science.

[167] Dice J.F., Terlecky S.R., Chiang H.L., Olson T.S., Isenman L.D., Short-Russell S.R., Freundlieb S., Terlecky L.J. (1990). A Selective Pathway for Degradation of Cytosolic Proteins by Lysosomes. Semin. Cell Biol..

[168] Kirchner P., BourdenxID M., Madrigal-MatuteID J., Tiano S., Diaz A., BartholdyID B.A., Will B., Maria CuervoID A., Bourdenx M., Madrigal-Matute J. (2019). Proteome-Wide Analysis of Chaperone-Mediated Autophagy Targeting Motifs. PLoS Biol..

[169] Bandyopadhyay U., Cuervo A.M. (2008). Entering the Lysosome through a Transient Gate by Chaperone-Mediated Autophagy. Autophagy.

[170] Bandyopadhyay U., Kaushik S., Varticovski L., Cuervo A.M. (2008). The Chaperone-Mediated Autophagy Receptor Organizes in Dynamic Protein Complexes at the Lysosomal Membrane. Mol. Cell Biol..

[171] Alvarez-Erviti L., Rodriguez-Oroz M.C., Cooper J.M., Caballero C., Ferrer I., Obeso J.A., Schapira A.H. (2010). V Chaperone-Mediated Autophagy Markers in Parkinson Disease Brains. Arch. Neurol..

[172] Murphy K.E., Gysbers A.M., Abbott S.K., Spiro A.S., Furuta A., Cooper A., Garner B., Kabuta T., Halliday G.M. (2015). Lysosomal-Associated Membrane Protein 2 Isoforms Are Differentially Affected in Early Parkinson’s Disease. Mov. Disord..

[173] Pang S., Chen D., Zhang A., Qin X., Yan B. (2012). Genetic Analysis of the LAMP-2 Gene Promoter in Patients with Sporadic Parkinson’s Disease. Neurosci. Lett..

[174] Sala G., Stefanoni G., Arosio A., Riva C., Melchionda L., Saracchi E., Fermi S., Brighina L., Ferrarese C. (2014). Reduced Expression of the Chaperone-Mediated Autophagy Carrier Hsc70 Protein in Lymphomonocytes of Patients with Parkinson’s Disease. Brain Res..

[175] Papagiannakis N., Xilouri M., Koros C., Simitsi A.-M., Stamelou M., Maniati M., Stefanis L. (2019). Autophagy Dysfunction in Peripheral Blood Mononuclear Cells of Parkinson’s Disease Patients. Neurosci. Lett..

[176] Cuervo A.M., Stafanis L., Fredenburg R., Lansbury P.T., Sulzer D. (2004). Impaired Degradation of Mutant α-Synuclein by Chaperone-Mediated Autophagy. Science.

[177] Martinez-Vicente M., Talloczy Z., Kaushik S., Massey A.C., Mazzulli J., Mosharov E.V., Hodara R., Fredenburg R., Wu D.C., Follenzi A. (2008). Dopamine-Modified α-Synuclein Blocks Chaperone-Mediated Autophagy. J. Clin. Invest.

[178] Mak S.K., McCormack A.L., Manning-Bog A.B., Cuervo A.M., Di Monte D. (2010). a Lysosomal Degradation of Alpha-Synuclein in Vivo. J. Biol. Chem..

[179] Vogiatzi T., Xilouri M., Vekrellis K., Stefanis L. (2008). Wild Type Alpha-Synuclein Is Degraded by Chaperone-Mediated Autophagy and Macroautophagy in Neuronal Cells. J. Biol. Chem..

[180] Xilouri M., Vogiatzi T., Vekrellis K., Park D., Stefanis L. (2009). Abberant Alpha-Synuclein Confers Toxicity to Neurons in Part through Inhibition of Chaperone-Mediated Autophagy. PLoS ONE.

[181] Xilouri M., Vogiatzi T., Vekrellis K., Stefanis L. (2008). Alpha-Synuclein Degradation by Autophagic Pathways: A Potential Key to Parkinson’s Disease Pathogenesis. Autophagy.

[182] Xilouri M., Brekk O.R., Kirik D., Stefanis L. (2013). LAMP2A as a Therapeutic Target in Parkinson Disease. Autophagy.

[183] Kabuta T., Setsuie R., Mitsui T., Kinugawa A., Sakurai M., Aoki S., Uchida K., Wada K. (2008). Aberrant Molecular Properties Shared by Familial Parkinson’s Disease-Associated Mutant UCH-L1 and Carbonyl-Modified UCH-L1. Hum. Mol. Genet..

[184] Orenstein S.J., Kuo S.-H., Tasset I., Arias E., Koga H., Fernandez-Carasa I., Cortes E., Honig L.S., Dauer W., Consiglio A. (2013). Interplay of LRRK2 with Chaperone-Mediated Autophagy. Nat. Neurosci..

